# The expression of CD123 can decrease with basophil activation: implications for the gating strategy of the basophil activation test

**DOI:** 10.1186/s13601-016-0100-4

**Published:** 2016-04-01

**Authors:** Alexandra F. Santos, Natalia Bécares, Alick Stephens, Victor Turcanu, Gideon Lack

**Affiliations:** Division of Asthma, Allergy and Lung Biology, Department of Pediatric Allergy, St Thomas’ Hospital, King’s College London, 2nd Floor, Stairwell B, South Wing, Westminster Bridge Road, London, SE1 7EH UK; MRC and Asthma UK Centre in Allergic Mechanisms of Asthma, London, UK; Immunoallergology Department, Coimbra University Hospital, Coimbra, Portugal; Gulbenkian Programme for Advanced Medical Education, Lisbon, Portugal

**Keywords:** Basophil activation test, Peanut allergy, CD123, CD63, CD203c, IL-3

## Abstract

**Background:**

Basophil activation test (BAT) reproduces IgE-mediated allergic reactions in vitro and has been used as a diagnostic test. Different markers can be used to identify basophils in whole blood and have implications for the outcome of the test. We aimed to assess changes in the expression of CD123 and HLA-DR following basophil activation and to select the best gating strategy for BAT using these markers.

**Methods:**

BAT was performed in whole blood from 116 children. Peanut extract, anti-IgE, anti-FcεRI or formyl-methionyl-leucyl-phenylalanin (fMLP) was used for stimulation. Surface expression of CD123, HLA-DR, CD63 and CD203c was evaluated by flow cytometry.

**Results:**

In some cases, gating on CD123+/HLA-DR− led to the loss-to-analysis of basophils in conditions where basophils were activated. Adding CD203c as an identification marker restored the cell number. Basophils remained HLA-DR-negative with activation. CD123 expression decreased following stimulation with fMLP (n = 116, p < 0.001), anti-IgE (n = 104, p < 0.001) and peanut (n = 42, p < 0.001). The decrease in the mean fluorescence intensity of CD123 correlated with the up-regulation of basophil activation markers, CD63 (r_s_ = −0.31, p < 0.001) and CD203c (r_s_ = −0.35, p < 0.001). BAT to peanut gating basophils on CD203c+/CD123+/HLA-DR− reduced the false-negatives (1 vs. 5 %) and showed a higher diagnostic accuracy compared to using CD123+/HLA-DR− (97 vs. 91 %). CD203c+ appeared as an alternative gating strategy allowing two-colour BAT.

**Conclusions:**

Basophils of a subset of patients down-regulate CD123 with activation. The use of CD203c before gating on CD123+/HLA-DR− cells or in isolation ensures the identification of the entire basophil population and accurate assessment of basophil activation, with important diagnostic implications.

## Background

The basophil activation test (BAT) is a flow cytometry-based assay that reproduces IgE-mediated allergic reactions in vitro. Following cell stimulation and activation, basophils undergo degranulation with the release of histamine, leukotrienes and cytokines, and up-regulate the expression of activation markers on their surface, such as CD63 and CD203c, which can be measured by flow cytometry [1, 2]. CD63 is a lysosomal-associated membrane protein (LAMP-3) and is not expressed on resting basophils but only after degranulation as the granules fuse with the plasma membrane. Its expression is bimodal, as only a subset of basophils express CD63, therefore it is usually represented as a proportion of positive cells (i.e. %CD63+ basophils). CD203c is constitutively and specifically expressed on the surface of resting basophils, which increases after basophil activation; thus, it can be used as an identification as well as an activation marker [3]. CD203c has been related to piecemeal degranulation and CD63 to anaphylactic degranulation [4, 5]. The BAT has been used primarily as a research tool and its application for clinical use in the diagnosis and monitoring of allergic diseases [6–10], namely following immuno-modulatory treatments such as allergen-specific immunotherapy [11–13] and omalizumab [14–16], is still in development. Our first allergen-specific (peanut) study that is clinically validated and based on double-blind-placebo-controlled-food-challenges (DBPCFC) initially established retrospectively diagnostic cut-off values for CD63 expression (n = 104) and verified this prospectively in a second population (n = 65) [6]. In this study [6], BAT showed 97 % accuracy in the diagnosis of peanut allergy. This enhanced diagnostic performance is in large part due to inclusion of CD203c in the gating strategy.

Different cell-surface markers may be preferred for the identification of basophils in whole blood. One of the commonly used is the combination of CD123 and HLA-DR [1, 2]. CD123 is the low affinity subunit of the IL-3 receptor and is highly expressed on plasmocytoid dendritic cells and basophils, and in low levels on monocytes, eosinophils, myeloid dendritic cells and hematologic progenitor cells. While eosinophils can be excluded by side-scatter, additional staining with anti-HLA-DR discriminates between HLA-DR-negative basophils and HLA-DR-positive dendritic cells and monocytes. To exclude hematologic progenitor cells, an additional marker specific for basophils such as CD203c could be used. In previous studies, the expression of CD123 and HLA-DR were shown to be stable with the atopic status of patients and following basophil activation [2, 17]. However, stimulation of the IL-3 receptor by IL-3 can increase the baseline expression of CD203c and possibly CD63 and maximise the up-regulation of CD63 upon basophil activation [4, 18]. Therefore, we hypothesized that CD123 expression could change in response to basophil activation. With increasing attention given to basophils in the coordination of adaptive immune responses and their possible role in antigen presentation [19–21], we considered that there could be an increase in the expression of HLA-DR by basophils following activation by allergen or other stimulants. Given the important implications for the gating strategy to be adopted for BAT in future studies, we sought to determine whether the expression of CD123 and HLA-DR remained unchanged with basophil activation and to select the best gating strategy using these markers.

## Methods

### Study population

Results of BAT to peanut performed in children attending our Pediatric Allergy clinic, aged from 5 months to 17 years, performed using the same methodology as part of two clinical studies [6, 10] were analyzed. Peanut allergy was diagnosed based on a positive oral peanut challenge or the combination of a recent clear history of one or more systemic reactions to peanut and a weal diameter on skin prick testing (SPT) ≥8 mm and/or serum peanut-specific IgE ≥15 KU_A_/l [22]. Peanut-tolerance was defined by a negative oral peanut challenge or the ability to eat an age-appropriate quantity of peanut regularly (as defined by a validated food-frequency questionnaire [23]) without developing any allergic symptoms. Ethical approval was obtained from the South East London Research Ethics Committee 2 and parents of all children signed written informed consent.

### Whole blood basophil activation test

Heparinized whole blood (100 µl) was stimulated for 30 min at 37 °C with peanut extract (0.1; 1; 10; 100; 1000 and 10,000 ng/ml, ALK Abelló, Horsholm, Denmark) diluted in Rosewell Park Memorial Institute (RPMI, GIBCO, Paisley, UK). Polyclonal goat IgG anti-human IgE (1 µg/ml, Sigma-Aldrich, Poole, UK) and monoclonal mouse anti-human FcɛRI (2.5 µg/ml, Ebioscience, San Diego, CA, USA) were used as IgE-mediated positive controls. Formyl-methionyl-leucyl-phenylalanine (1 µM, fMLP, Sigma-Aldrich) was used as a non-IgE-mediated positive control (as it acts via a G-protein-coupled receptor (FPR-1) that activates MAPK pathways and phospholipase C bypassing part of the signalling pathway downstream the IgE receptor FcεRI). RPMI alone was used as a negative control. The reaction was stopped by adding cold ethylenediaminetetraacetic acid. Prior to erythrocyte lysis with BD Pharmlyse (BD Biosciences, San Jose, CA, USA), cells were stained with anti-CD123-fluorescein isothiocyanate (FITC) (Ebioscience), anti-CD203c-phyco-erythrin (PE), anti-HLA-DR-peridinin chlorophyll protein (PerCP) and anti-CD63-allophycocyanin (APC) (Biolegend, San Diego, CA, USA) for 30 min. In selected experiments, anti-CD14-PECy7, anti-CD3-pacific blue (PB), anti-CD19-PB, anti-CD41-PB and anti-CD56-PB (Biolegend) were additionally used. The surface expression of these markers was evaluated using FACS CantoII with FACSDiva software (BD Biosciences, San Jose, CA, USA). Data was analysed with FlowJo software version 7.6.1 (TreeStar, Ashland, OR, USA).

Basophil activation was expressed as a proportion of CD63+ basophils, corrected for the negative control, and as a ratio of the MFI of CD203c-PE of stimulated to unstimulated basophils, the stimulation index of CD203c (SI CD203c). The variation of CD123 was defined as the proportion of the difference between the MFI of CD123-FITC of the negative control and of the stimulated cells and the MFI of CD123-FITC of the negative control, and was calculated using the formula (MFI CD123-FITC of negative control − MFI CD123-FITC of stimulated cells)/MFI CD123-FITC of negative control. When assessing basophil activation induced by anti-IgE (n = 104), patients with non-responder basophils, i.e. basophils which did not respond to any IgE-mediated stimulants but only to fMLP, were excluded. When evaluating the response to peanut, peanut allergic patients with responding-basophils were considered (n = 42). In the data analysis, when only one concentration of peanut extract was used, 100 ng/ml was selected unless indicated otherwise.

### Statistical analysis

Qualitative variables were represented as number of patients and percentage (taking into account the missing values) and groups were compared using the Fisher’s exact test or Chi square test, as appropriate. Continuous variables were represented as median and range and were compared using the Mann–Whitney U test or Kruskall–Wallis test, as appropriate. Wilcoxon-signed rank test was used to compare samples before and after stimulation.

For receiver-operating characteristic (ROC) curve analysis, the performance of the average percentage of CD63-positive basophils at 10 and 100 ng/ml of peanut extract determined using different gating strategies was evaluated against the patients’ allergic status to peanut, i.e. in relation to allergy versus tolerance.

All statistical analyses were performed with SPSS 20.0 for Windows. Significance was determined using a two-sided α level of 0.05.

## Results

### Gating on basophils using CD123 and HLA-DR led to the loss of cells

**Fig. 1:**
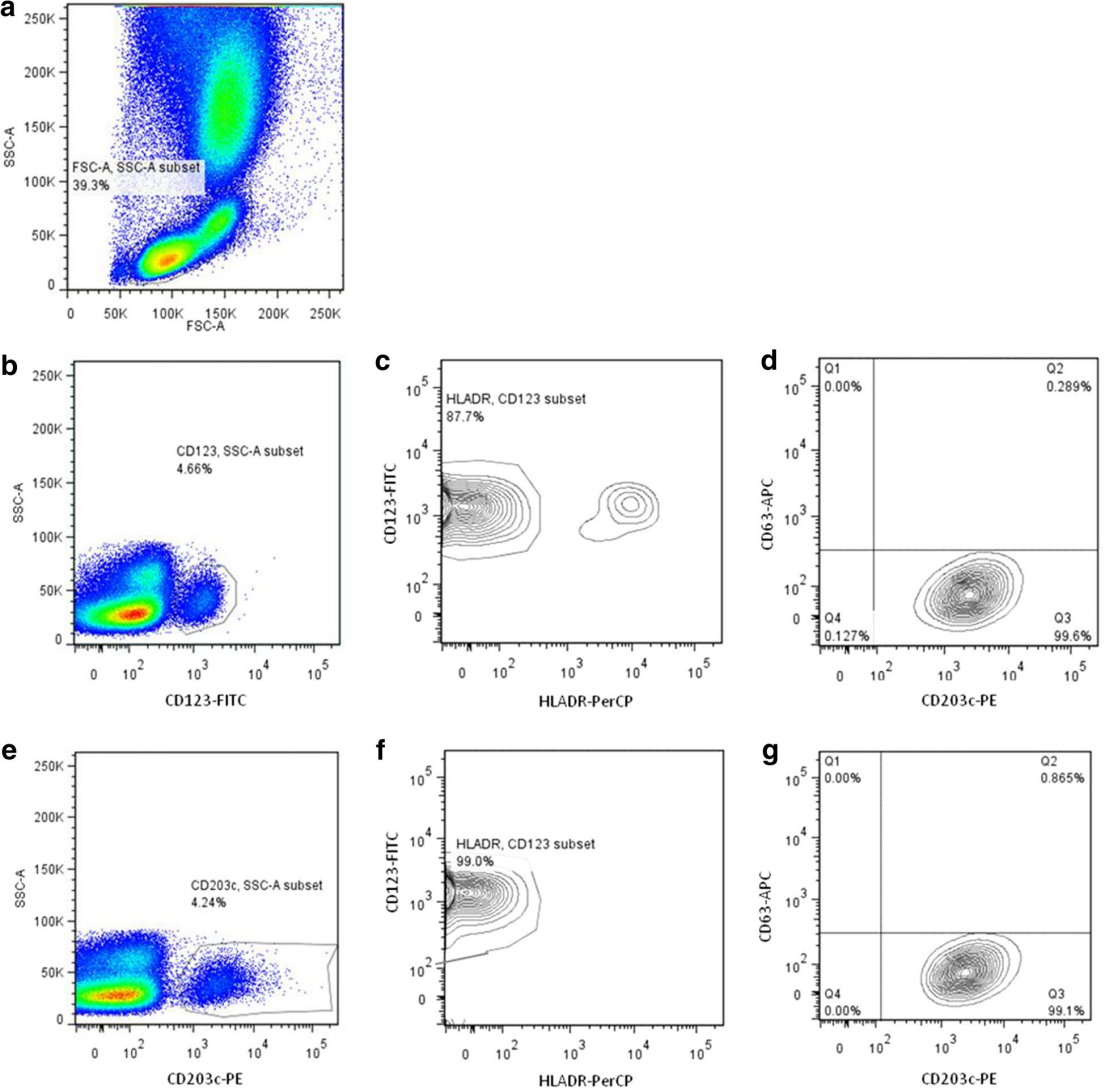
Basophils were identified in whole blood in the lymphocyte-monocyte area (**a**) as SSC^low^/CD123+/HLA-DR− (**b**, **c**) or as SSC^low^/CD203c+ (**e**) or as SSC^low^/CD203c+/CD123+/HLA-DR− (**e**, **f**) cells. CD63 expression was assessed in basophils identified as SSC^low^/CD123+/HLA-DR− (**d**) or SSC^low^/CD203c+/CD123+/HLA-DR− (**g**).

Identifying basophils with CD123 and HLA-DR (Fig. 1a–d) led to the loss to analysis of cells particularly in conditions where basophils were activated. The baseline number of unstimulated basophils was variable (median = 1721, IQR = 1225–2184) but comparable between atopic and non atopic patients (p = 0.444) and between peanut allergic and peanut tolerant children (p = 0.739). Following basophil stimulation with fMLP (p = 0.012) and anti-IgE (p = 0.005), the number of basophils was significantly reduced compared to baseline (Table 1).

**Table 1:** Number of basophils (gated as SSClow/CD123+/HLA-DR− cells) and expression of CD123 on the surface of basophils (gated as SSClow/CD203c+ cells) as measured by MFI of CD123-FITC in different stimulation conditions

Stimulant	n	Pre-stimulation	Post-stimulation	p value
Number of basophils				
fMLP	116	1722 (1226, 2184)	1572 (1037, 2100)	*0.012*
Anti-IgE	104	1722 (1221, 2191)	1414 (972, 1995)	*0.005*
Peanut extract	42	1732 (1212, 2174)	1514 (914, 2041)	0.134
MFI of CD123-FITC				
fMLP	116	1082 (98, 1549)	514 (75, 1020)	<*0.001*
Anti-IgE	104	1081 (95, 1549)	495 (60, 1111)	<*0.001*
Peanut extract	42	1172 (80–2058)	293 (40, 1244)	<*0.001*

Median (inter-quartile range) is represented. p value refers to the comparison of post-stimulation conditions with the negative control

**Fig. 2:**
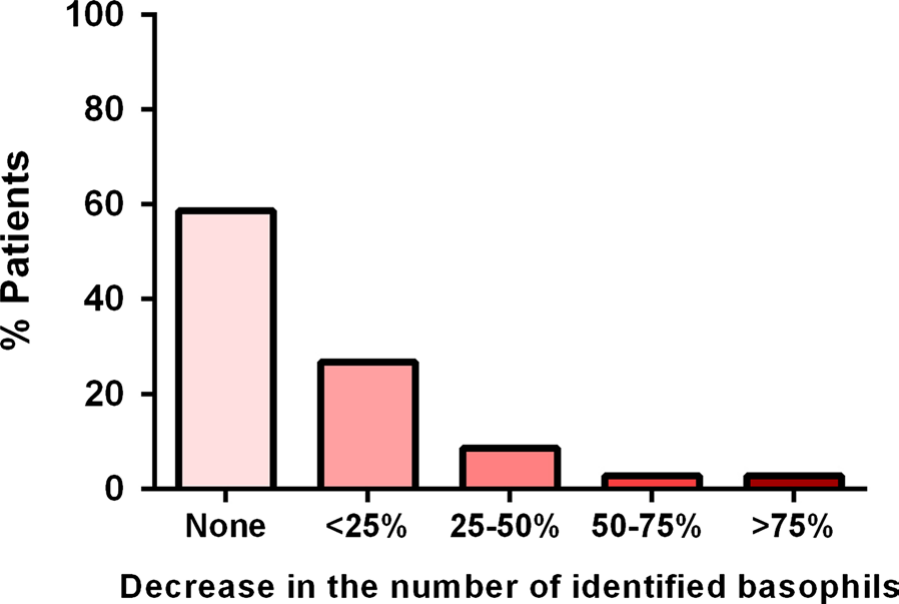
Variation in the number of identified basophils following anti-IgE stimulation compared to the negative control (n = 104). Variation was calculated as (number of identified basophils pre-stimulation − number of identified basophils post-stimulation)/number of identified basophils pre-stimulation

Of note, if only patients with non-responder basophils were considered (n = 12), the change in basophil number was significant after fMLP stimulation (p = 0.004) but not after anti-IgE stimulation (p = 0.099), suggesting that the reduction in cell number was dependent on basophil activation. Considering anti-IgE stimulation, 14 % of patients showed more than 25 % decrease in the number of basophils compared to the negative control (Fig. 2).

In 27 % of patients, this number decreased to below 1000 basophils (the minimum number of cells usually required for BAT). Selecting peanut allergic patients, a trend was seen toward a reduction in the basophil number after stimulation with 1000 ng/ml of peanut extract compared to the negative control (n = 42, p = 0.081). As the starting volume of blood, and thus the starting number of cells, was similar in all experimental conditions, we hypothesized that the expression of the identification markers, CD123 and/or HLA-DR, changed with basophil activation.

### CD123 is down-regulated with basophil activation

**Fig. 3:**
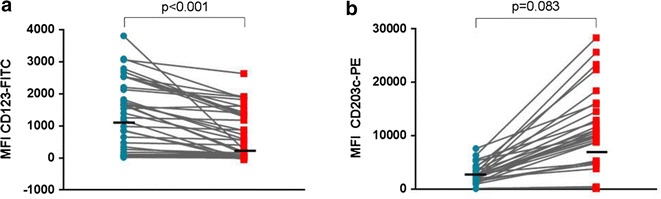
Changes in the MFI of CD123-FITC (**a**) and in the MFI of CD203c-PE (**b**), following stimulation with 100 ng/ml of peanut extract (n = 42 peanut allergic patients)

**Fig. 4:**
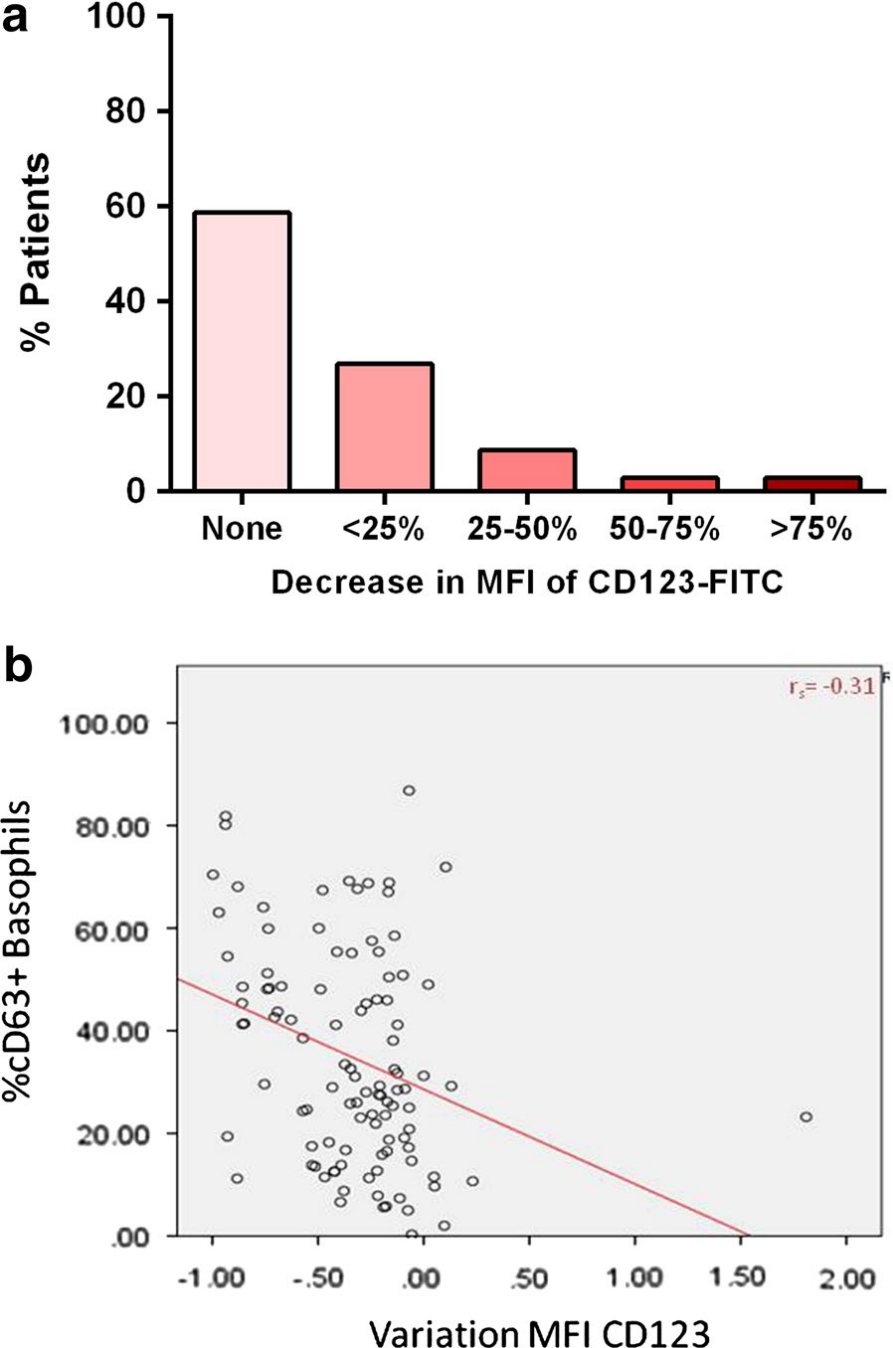
Down-regulation of CD123 expression with basophil activation. **a** Change in the MFI of CD123-FITC on the surface of basophils following anti-IgE stimulation. **b** Correlation between decrease in CD123 and up-regulation of CD63 on the surface of basophils

To evaluate the changes in the expression of CD123 and HLA-DR on the surface of basophils, we used CD203c to gate on the basophil population (Fig. 1a–g). The baseline MFI of CD123-FITC was variable between patients (median = 1082, IQR = 98–1549) but comparable between atopic and non-atopic children (p = 0.153) and between peanut allergic and peanut tolerant patients (p = 0.826). Down-regulation of CD123 by basophils was seen following stimulation with fMLP (n = 116, p < 0.001), anti-IgE (n = 104, p < 0.001) and peanut extract (n = 42 peanut allergic patients, p < 0.001)—Table 1 and Fig. 3a. In 92.3 % of patients, anti-IgE stimulation led to a decrease in the MFI of CD123-FITC compared to the negative control: in 38.5 less than 25 % decrease, in 26.9 % between 25 and 50 % decrease, in 13.5 % between 50 and 75 % decrease and in 13.5 more than 75 % decrease (Fig. 4a). The down-regulation of CD123 expression on the surface of basophils stimulated by fMLP and by anti-IgE was correlated (r_s_ = 0.723, p < 0.001), suggesting this phenomenon happened in the same patients with different stimulants. The decrease in CD123 expression with anti-IgE stimulation was similar between atopic and non atopic (p = 0.828) and between peanut allergic and peanut tolerant children (p = 0.431). The expression of CD123 was stable when basophils were not activated—for example, after stimulation with peanut in peanut tolerant patients (p = 0.658) or after stimulation with anti-IgE in non-responders’ basophils (p = 0.083), while down-regulation was still observed in this subgroup after stimulation with fMLP (p = 0.006).

Basophils were HLA-DR-negative and were distinct from the CD123+ HLA-DR+ population of plasmacytoid dendritic cells. Following basophil activation, the HLA-DR expression did not increase and remained distinct from HLA-DR+ cells.

### Decrease in CD123 is correlated with the up-regulation of CD63 and CD203c

Taken together the previous observations indicate that the down-regulation of CD123 by basophils is an activation-dependent phenomenon. A weak statistically significant correlation was observed between the decrease in the MFI of CD123 and the up-regulation of basophil activation markers after stimulation with anti-IgE (Fig. 4b) as measured by the stimulation index of CD203c (r_s_ = −0.35, p < 0.001) or by the percentage of CD63-positive basophils (r_s_ = −0.31, p < 0.001), suggesting that the basophils that down-regulate CD123 the most are also the ones that express more CD63 and CD203c. The correlations between changes in the MFI of CD123-FITC and in the percentage of CD63+ basophils or the SI of CD203c following stimulation with fMLP (−0.191, p = 0.04 and −0.223, p = 0.016, respectively) and following stimulation with peanut extract (−0.229, p = 0.145 and −0.31, p = 0.051, respectively) were less strong.

### Additional use of CD203c prevented the loss-to-analysis of activated basophils

**Fig. 5:**
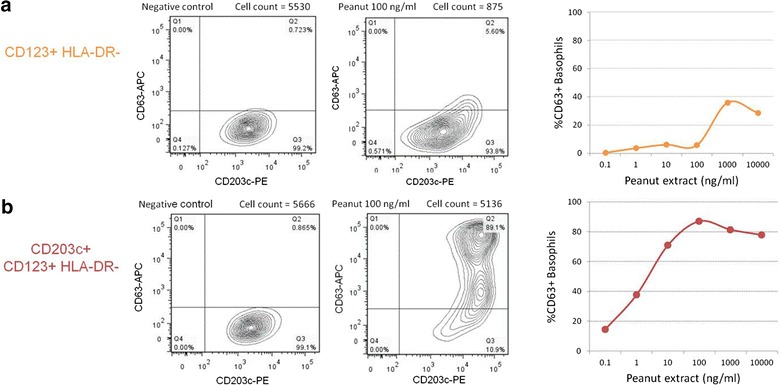
Gating with CD123/HLA-DR led to the loss-to-analysis of basophils and underestimation of basophil activation. In a representative experiment, basophils were gated as SSClow/CD123^bright^/HLA-DR− (**a**) or SSC^low^/CD203c+/CD123+/HLA-DR− (**b**). The percentage of CD63+ basophils is represented in different conditions: negative control, peanut extract 100 ng/ml and peanut-dose–response curve. Note the bi-exponential display [38]

The down-regulation of CD123 with basophil activation has important implications in gating strategies that depend on CD123. Identifying basophils using CD123 and HLA-DR led to the loss-to-analysis of basophils, particularly of the ones with higher expression of the activation markers CD63 and CD203c, and thus leading to an underestimation of basophil activation (Table 2; Fig. 5a). Adding CD203c as an identification marker restored the cell number, regardless of the basophil activation status and allowed to include the basophils that were activated the most in the analysis, improving the outcome of the test (Table 2; Fig. 5b). The expression of CD203c remained stable or increased following basophil activation, allowing a good separation from the remaining blood cells (Fig. 3b).

**Table 2:** Comparison of different strategies to gate on basophils

Parameters	Stimulants	CD123+ HLA-DR−	CD203c+	CD203c+ CD123+ HLA-DR−	Overall p value	p value^1^	p value^2^	p value^3^
Number of basophils	Negative control^a^	1722 (1226, 2184)	1782 (1334–2239)	1697 (1268–2134)	0.635	0.390	0.436	0.908
	Anti-IgE^b^	1414 (972–1995)	2156 (1620–3097)	1939 (1452–2877)	<*0.001*	<*0.001*	0.06	<*0.001*
	fMLP^a^	1572 (1037, 2100)	2146 (1684–2940)	1891 (1447–2715)	<*0.001*	<*0.001*	0.084	<*0.001*
	Peanut extract^c^	1514 (914–2041)	2351 (1748–3062)	2104 (1615–2752)	<*0.001*	<*0.001*	0.348	*0.001*
%CD63 + basophils	Anti-IgE^b^	24.8 (10.7, 42.5)	29.4 (17.3–48.9)	32.0 (17.1–53.9)	*0.021*	*0.020*	0.809	*0.013*
	fMLP^a^	28.2 (15.9, 42.3)	41.1 (26.3–53.4)	41.4 (26.9–56.9)	<*0.001*	<*0.001*	0.644	<*0.001*
	Peanut extract^c^	32.3 (10.9, 56.2)	41.0 (18.0–56.9)	42.1 (20.0–68.5)	0.185	0.220	0.561	0.074
MFI CD63	Anti-IgE^b^	104.0 (26.3–195.2)	148 (36, 345)	159.3 (45.4–350.8)	*0.019*	*0.029*	0.611	*0.009*
	fMLP^a^	139 (37, 284)	285 (76, 683)	307 (81, 708)	<*0.001*	<*0.001*	0.678	<*0.001*
	Peanut extract^c^	138.4 (24.8–270.7)	190 (37, 513)	202.9 (42.6–567.4)	0.267	0.271	0.629	0.113
SI CD203c	Anti-IgE^b^	2.4 (1.5–3.8)	2.9 (2.0–4.2)	3.2 (2.0–4.6)	*0.004*	*0.028*	0.191	*0.002*
	fMLP^a^	2.3 (1.7–3.1)	2.6 (2.2–3.7)	2.8 (2.2–4.1)	<*0.001*	*0.001*	0.280	<*0.001*
	Peanut extract^c^	3.3 (1.8–5.3)	3.8 (2.4–4.8)	4.3 (2.4–5.4)	0.193	0.386	0.260	0.088
MFI CD203c	Anti-IgE^b^	2331 (197–5633)	4833 (254, 9020)	5013 (29–9161)	*0.008*	*0.011*	0.677	*0.005*
	fMLP^a^	2763 (216–5139)	4549 (279, 7961)	4908 (334–8270)	*0.001*	*0.002*	0.813	*0.001*
	Peanut extract^c^	3631.6 (179.4–8491.4)	5312 (206, 11,976)	7012 (236–11,857)	0.173	0.14	0.714	0.083

Median and inter-quartile range are represented. Medians were compared between the three groups using Kruskall–Wallis test and between two groups using Mann–Whitney U test

^a^n = 116

^b^n = 104 (non-responders were excluded)

^c^n = 42 (peanut allergic)

^1^CD123+/HLA-DR− versus CD203c+

^2^CD203c+ versus CD203c+/CD123+/HLA-DR−

^3^CD123+/HLA-DR− versus CD203c+/CD123+/HLA-DR−

### Selecting the optimal gating strategy using CD203c

In order to select the best gating strategy using the available markers, we compared the results of the BAT following the identification of basophils as SSC^low^/CD203c+ cells, as SSC^low^/CD123+/HLA-DR− cells or as SSC^low^/CD203c+/CD123+/HLA-DR−. To combine the three identification markers to gate on basophils, we first selected the SSC^low^/CD203c+ cells and then gated on the plot CD123/HLA-DR following the contour of the cell population of interest, including CD123^low^ as well as CD123^high^ cells, all HLA-DR− (Fig. 1f).

The gating strategy using CD203c+/CD123+/HLA-DR− proved superior to CD123+/HLA-DR− and similar to what was observed for gating on CD203c+ cells alone (Table 2). The combination of the three markers was superior to CD203c+ in the subgroup of peanut allergic patients improving the detection of basophil activation (Table 2, borderline non-significant p values for %CD63+ basophils, SI CD203c and MFI of CD203c) and in patients with a subset of CD203c+/HLA-DR+ cells thus avoiding contamination with HLA-DR+ cells. However, in the majority of patients, the gating strategies SSC^low^/CD203c+ and SSC^low^/CD203c+/CD123+/HLA-DR− were comparable. SSC^low^/CD203c+ could be used as an alternative gating strategy in a two-colour BAT.

In additional experiments (n = 10) using antibodies anti-CD14, anti-CD3, anti-CD19, anti-CD41 and in six of these experiments also anti-CD56, there was no contamination of the gating strategy using SSC^low^/CD203c+/CD123+/HLA-DR− with other immune cells nor did they express CD203c. Only in one patient, CD14+ cells were also CD203^low^ and HLA-DR+. In the PB-positive (PB+) population, there was a minor expression of CD63, possibly by CD41+ platelets, but this population was gated out as it did neither express CD123 nor CD203c, and CD63 expression remained stable with activation (data not shown).

### Gating strategy of BAT has important diagnostic implications

**Fig. 6:**
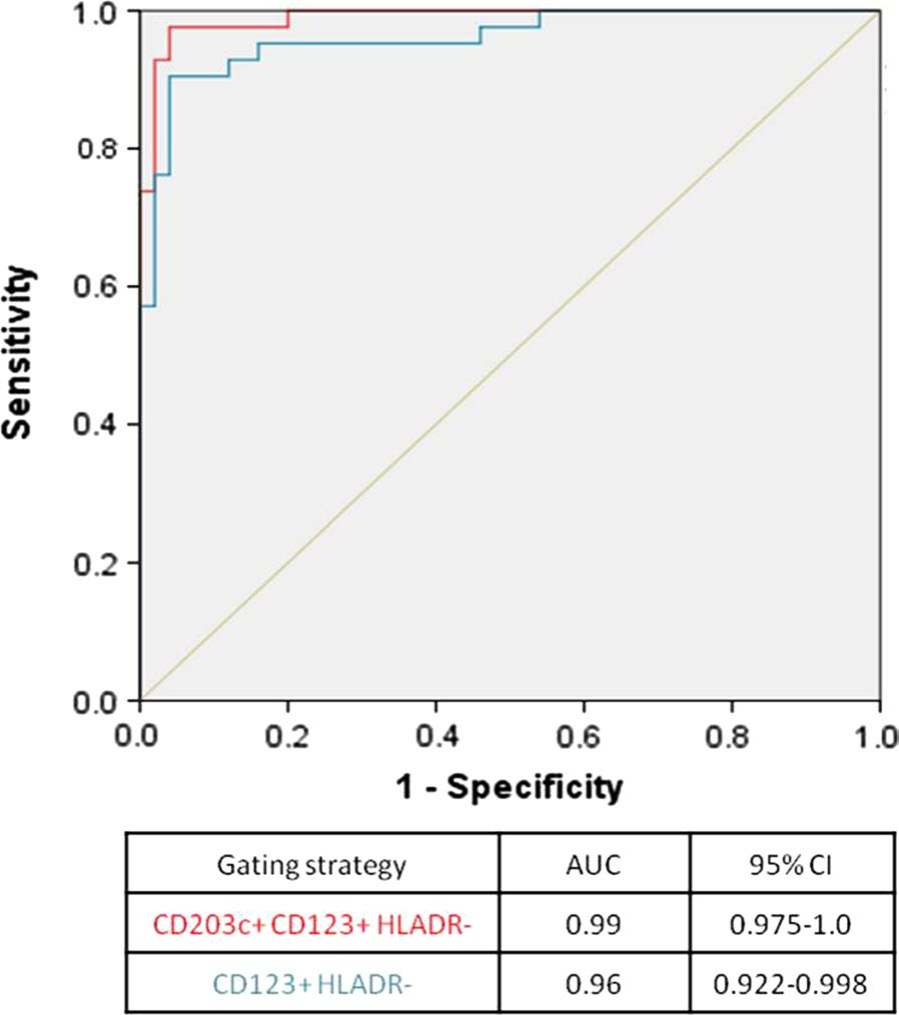
ROC curve of the average %CD63+ basophils at 10 and 100 ng/ml of peanut extract using different basophil gating strategies: SSC^low^/CD203c+/CD123+/HLA-DR− (*red*), SSC^low^/CD123+/HLA-DR− (*blue*)

The diagnostic performance of BAT gating on SSC^low^/CD203c+/CD123+/HLA-DR− was superior to the one using SSC^low^/CD123+/HLA-DR− with a larger area under the ROC curve (Fig. 6). The optimal cut-off based on the ROC curve generated using the latter gating strategy resulted in a 91 % diagnostic accuracy with 5 % false-negatives and 3 % false positives (Table 3). Adopting the SSC^low^/CD203c+/CD123+/HLA-DR− gating strategy resulted in a diagnostic accuracy of 97 %, as we recently reported [6], with 1 % false-negatives and 2 % false-positives. Unusually, this methodological improvement resulted in both enhanced sensitivity and specificity of BAT in the diagnosis of peanut allergy. Figure 5 shows an example of a patient that would be considered false negative if gating was confined to CD123+/HLADR− cells. Furthermore, using this gating strategy, 15 % of patients showed <500 basophils in at least one condition and thus BAT would be uninterpretable.

**Table 3:** Diagnostic accuracy of the basophil activation test to peanut using different gating strategies

	Gating strategy	
	CD123+ HLADR−	CD203c+ CD123+ HLADR−
Optimal cut-off	6.8	8.1
AUC ROC for the cut-off	0.91	0.97
Accuracy (%)	91	97
Sensitivity (%)	88	98
Specificity (%)	94	96
PPV (%)	93	95
NPV (%)	90	98
%True positives	40	45
%False positives	3	2
%True negatives	51	52
%False negatives	5	1
LR+	14.7	24.4
LR−	0.13	0.02

The optimal cut-off was determined for the %CD63+ basophils following stimulation with 100 ng/ml of peanut extract

*PPV* positive predictive value, *NPV* negative predictive value, *LR*+ positive likelihood ratio, *LR*− negative likelihood ratio

## Discussion

BAT can be used to diagnose allergic disease and to study the underlying immunological mechanisms. The methodology of identification of basophils has important consequences for the outcome of the test. In this study, we showed that CD123 is down-regulated with basophil activation and gating strategies that depend solely on this marker lead to the loss-to-analysis of activated basophils and to the underestimation of basophil activation. Using CD203c in addition to CD123/HLA-DR or in isolation proved superior to gating on CD123/HLA-DR, reducing the number of false-negatives and false-positives and improving the diagnostic accuracy from 91 to 97 %. When BAT is used as an allergy test, the gating strategy adopted has diagnostic implications in the assessment of individual patients. Basophils have been identified with CD123 and HLA-DR in previous studies, using flow cytometry and other techniques [24], and CD123 expression was reported to be stable with the atopic status of the patient and following basophil activation, although no direct comparisons were made [2, 17, 25]. In our study, the MFI of CD123-FITC was comparable between atopic and non atopic patients, but there was a decrease in the MFI of CD123-FITC with basophil activation. This is in contrast to previous studies [2, 17, 25] and is probably related to differences in the study population (e.g. adults vs. children), in the disease models studied (e.g. patients with respiratory vs. food allergies) and in the study design (e.g. basophils stimulated with allergen in vivo vs. in vitro, which can have different kinetics). We do not believe compensation played a role in the observed changes as this phenomenon only happened in a subset of patients and in other patients the expression of CD123 was stable regardless of the increase in the expression of CD63 and CD203c. Furthermore, while the spectrum of CD203c-PE overlapped with CD123-FITC, the spectra of CD123-FITC and of CD63-APC did not overlap and we performed compensation before running the samples, used the same compensation matrix during acquisition of all samples for each patient and did not see any differences in compensation within the same experiment. Although the size and the granularity of basophils change with activation and degranulation [5, 26], these modifications do not lead to cell loss in the FSC/SSC gate and basophil numbers are stable with and without stimulation using identification markers such as CD203c. With respect to HLA-DR, basophils were HLA-DR-negative and kept distinct of HLA-DR-positive cells in all conditions. These findings are consistent with previous reports [17] and with recent studies that did not confirm a role for basophils in antigen-presentation in humans [27, 28].

The expression of CD123 being dependent on basophil activation raises the question as to whether CD123 can be used as a basophil activation marker. However, this phenomenon is significant in only a subset of patients and the dose–response with increasing concentrations of the stimulant is subtle; thus, CD123 does not seem to offer any advantage to the existing activation markers, such as CD63 and CD203c.

The combination of the three identification markers, CD203c/CD123/HLA-DR, proved superior to using CD123/HLA-DR. We compared the diagnostic performance of BAT to peanut identifying basophils as CD123+/HLA-DR− cells and as CD203c+/CD123+/HLA-DR− cells and the latter resulted in a greater area under the ROC curve (0.96 vs. 0.99) and improved diagnostic accuracy (91 vs. 97 %). Furthermore, if we took into consideration a minimum number of basophils as exclusion criteria, 15 % of patients would be inevaluable using CD123+/HLADR−. The consequences of the gating strategy adopted are clinically relevant as the purpose of the BAT is to diagnose peanut allergy in individual patients. An example is illustrated in Fig. 5, where with basophils gated as CD123+/HLA-DR−, BAT would be considered negative at the diagnostic concentration of 100 ng/ml but in fact it was clearly positive when basophils were selected using the three markers’ strategy. This would be a false-negative with potential serious consequences, as it could lead to liberalization of peanut consumption with the risk of allergic reactions that are potentially severe. The loss-to-analysis of cells and/or the underestimation of basophil activation have important implications for the final outcome of the test and thus for the diagnosis of individual patients. The same applies to other clinical applications, such as monitoring of treatment, and to mechanistic experiments. The fact that we detected no contamination of the gating with other immune cells suggests that there would be no advantage in using a lineage negative antibody mixture to exclude other immune cells before gating on the CD203c+/CD123+/HLA-DR− cells as basophils.

Identifying basophils with CD203c alone lead to comparable outcome in terms of number of basophils and basophil activation markers to using CD203c+/CD123+/HLA-DR− and was also superior to using CD123+/HLA-DR−, as represented in Table 2. CD203c is a basophil specific marker in whole blood. Its constitutive expression is increased in patients with atopic eczema and food allergy [29–31], as previously described in terms of histamine release [32] suggesting it is a marker of underlying basophil activation, possibly reflecting ongoing piecemeal degranulation. This enhances the separation of CD203c+ basophils from the other blood cells in populations of highly atopic children [6]. The majority of patients in our study had eczema and other food allergies in addition to suspected peanut allergy and this represents a population where CD203c would be constitutively expressed at a higher level than in other children allowing a clear differentiation between CD203c+ basophils and other blood cells. The conjugation to the bright fluorochrome PE may have also contributed to the good identification of cells expressing CD203c. SSC^low^/CD203c+ is an alternative basophil identification strategy that has the advantage of serving also as an activation marker, enabling BAT to be performed as a two-colour (preferred) or even as a single colour BAT, which would make BAT easier and less expensive to perform.

This is the largest study looking at different strategies for identifying basophils using CD123 and its clinical implications and it is the first study to report the down-regulation of CD123 with basophil activation. The performance of BAT soon after blood collection and the use of live cells for flow cytometry on the same day contributed to the quality of the results. However, our study population was constituted mostly by highly atopic children enrolled in a study examining the use of BAT in the diagnosis of peanut allergy [6]. Therefore, our results may not apply to other populations, namely of non atopic patients or older patients being assessed for other conditions such as drug allergy. While assessing changes in basophil identification markers, we discovered that in a subset of patients basophils down-regulate CD123, the low affinity subunit of the IL-3 receptor, with basophil activation. This phenomenon seems patient-specific rather than specific for atopic or allergic status. In the patients where this is observed, the down-regulation happens only in conditions where basophils are activated and correlates with the degree of activation, as expressed by CD63 and CD203c. It is possible that the patients who showed higher down-regulation of CD123 after basophil activation are the ones with most severe clinical reactions but this remains to be confirmed. IL-3 is predominantly produced by T cells and is able to induce basophils to release histamine and up-regulate CD203c and CD63 in the absence of allergen [33, 34]. It can also act synergistically with allergen or other stimulants to increase basophil activation and histamine release [4, 18, 33]. The basophil intracellular pathways down-stream of the IL-3 and the IgE receptors seem indeed closely connected [34]. The response to this priming effect is variable between basophil donors and requires different concentrations of IL-3 [35]. Some research groups have used exogenous IL-3 to prime basophils in the BAT [4, 18, 36]. However, basophils secrete IL-3 themselves in response to IgE-mediated activation for autocrine priming, which has been suggested to be a possible mechanism underlying the hyper-reactive nature of the basophils of allergic patients [37]. We hypothesize that the down-regulation of CD123, which is part of the IL-3 receptor, could result from a basophil regulatory mechanism to avoid further cell activation, but this deserves further research.

## Conclusions

Basophils down-regulate CD123 with activation in a subset of patients and this can have significant deleterious diagnostic implications. While performing the BAT, the use of gating strategies that depend solely on CD123 may lead to loss-to-analysis of basophils, particularly of the ones that highly express CD63 and CD203c, resulting in a false-negative outcome for the test. To overcome this limitation, additional use of CD203c, both identification and activation marker, prevents the loss-to-analysis of activated basophils and allows accurate assessment of basophil activation and, consequently, a more accurate diagnosis of allergy.

## Abbreviations

APC: allophycocyanin
BAT: basophil activation test
DBPCFC: double-blind-placebo-controlled-food-challenge
FITC: fluorescein isothiocyanate
fMLP: formyl-methionyl-leucyl-phenylalanine
LAMP: lysosomal-associated membrane protein
PE: phycoerythrin
PerCP: peridinin chlorophyll protein
ROC: receiver-operating characteristic
RPMI: Rosewell Park Memorial Institute medium
SI: stimulation index
SPT: skin prick test
